# Deciphering the role of myofibroblasts in microvascular invasion of hepatocellular carcinoma

**DOI:** 10.3389/fphar.2025.1596181

**Published:** 2025-08-29

**Authors:** Xiang Liu, Jiangyang Pan, Lijia Wang, Qiao Xie, Tianbo Fan, Qi Wang

**Affiliations:** Department of Radiology, The Fourth Hospital of Hebei Medical University, Shijiazhuang, China

**Keywords:** Hepatocellular carcinoma, microvascular invasion, single-cell RNA sequencing analysis, myofibroblasts, intercellular communication, cell differentiation, prognostic markers

## Abstract

**Background:**

Hepatocellular carcinoma (HCC) often exhibits microvascular invasion (MVI), a feature with unclear mechanisms. Therefore, it is crucial to resolve its related cellular populations and molecular networks using single-cell analysis.

**Methods:**

Both single-cell RNA sequencing (scRNA-seq) and RNA-seq data for HCC were obtained from public databases. ScRNA-seq data were clustered and annotated using Seurat. DAVID, CellChat, and Monocle 2 were used for scRNA-seq functional enrichment analysis, intercellular communication analysis, and cell trajectory analysis, respectively. We further assessed key gene expression in HCC cell lines and examined their effects on cell functions using CCK-8, scratch, and transwell assays.

**Results:**

The HCC ecosystem comprising myofibroblasts (MFs), hepatocytes, proliferative hepatocytes, endothelial cells, dendritic cells, proliferative NK/T cells, plasma B cells, and macrophages was revealed. MFs showed the greatest difference between MVI-absent and MVI-present patients and were subdivided into five clusters. Key genes for angiogenesis are overexpressed in MF2 cells and enriched in the pathways of angiogenesis, cell migration, cell proliferation, and signal transduction. Pseudotime analysis revealed MF2 cells from MVI-present patients clustered at the terminal state and positively correlated with angiogenesis. *CAMK2N1* in the markers of MF2 cells was significantly associated with advanced M stage and poor prognosis. Further cellular assays showed that *CAMK2N1* expression was downregulated in HCC cells, and its knockdown increased the proliferation, migration, and invasion levels of cancer cells.

**Conclusion:**

This study highlighted the role and potential mechanism of MFs in promoting MVI formation and provides a potential marker for HCC prognosis among MF markers.

## Introduction

Hepatocellular carcinoma (HCC) is one of the most prevalent and lethal causes of cancer-related death worldwide (Chandarana et al., 2024; Wang et al., 2023). Risk factors associated with HCC include hepatitis B and C infections, non-alcoholic fatty liver disease, and alcoholic liver disease. Regardless of the underlying cause, the end result is liver fibrosis and cirrhosis, which eventually progress to cancer (Alawyia and Constantinou, 2023; Bansal et al., 2024). In recent years, anti-angiogenic therapies such as sorafenib, lenvatinib, and bevacizumab combined with atezolizumab have been widely used in advanced HCC, targeting VEGF/VEGFR and other angiogenic pathways to inhibit tumor vascularization (Finn et al., 2020; Kudo et al., 2019; Bruix et al., 2017). However, clinical resistance is common, driven by tumor heterogeneity, compensatory angiogenic signaling, and immune microenvironment adaptation (Federico et al., 2022). Moreover, the lack of reliable biomarkers and limited understanding of stromal cell-mediated angiogenesis restrict therapeutic precision (Tao et al., 2020). These limitations highlight the need to explore cellular mechanisms underlying angiogenesis in HCC, especially those contributing to microvascular invasion (MVI), which is closely linked to poor prognosis.

HCC, as a multi-vascular malignancy, frequently exhibits histological features of MVI (Sun et al., 2023; Cao et al., 2023). MVI includes a variety of microvascular structures, including small thin-walled vessels in the tumor capsule or adjacent fibrotic nonneoplastic liver, and thicker muscularized vessels as peripheral branches of portal veins, hepatic arteries, or hepatic veins (Hwang et al., 2023; Gouw et al., 2011). Clinical MVI imaging showed capsule rupture, irregular tumor margins, peritumoral enhancement, multifocal tumors, increased tumor size, and increased glucose metabolism on positron emission tomography-computed tomography (Unal et al., 2016). A systematic review study showed that the prevalence of MVI in HCC patients ranged from 15% to 57.1% (Rodriguez-Peralvarez et al., 2013). MVI has been clinically recognized as a prognostic factor for HCC after surgical treatment (Zhou et al., 2022). It should be noted, however, that the prognostic value of MVI varies with invasiveness (Nitta et al., 2019). In addition, the lack of definition and grading of MVI and reported inter-observer or intra-observer differences lead to great heterogeneity in the evaluation of this histological feature of HCC (Rodriguez-Peralvarez et al., 2013). Therefore, there is an urgent need to clarify this issue.

Single-cell analysis is promising as a means of understanding the heterogeneity of this unique histological feature and its relationship with HCC disease development (Xiao et al., 2019; Zulibiya et al., 2023). Currently, researchers have found, based on single-cell analysis, that tumor cells can reprogram CD10+ALPL + neutrophils through the NAMPT-NTRK1 signaling axis, leading to immune resistance and HCC progression (Meng et al., 2023). In this study, we analyzed the single-cell RNA sequencing (scRNA-seq) data of patients with MVI present and two patients with MVI absent to reveal the complex cell population of HCC patients with MVI present, the protagonist population of myofibroblasts promoting MVI, and the molecular network that interacts with hepatocytes. Finally, the markers of the main population of myofibroblasts promoting MVI were subjected to RNA-seq analysis from the The Cancer Genome Atlas (TCGA) dataset to identify candidate targets that may be potentially indicative of HCC prognosis. This approach enables the resolution of cellular heterogeneity that is often masked in bulk tissue analysis, allowing for the identification of rare but functionally significant cell subsets involved in MVI. By mapping cell-specific gene expression and trajectory, single-cell analysis provides a precise framework for decoding histological complexity in HCC.

## Materials and methods

### Searching for and preprocessing scRNA-seq data for human HCC

ScRNA-seq data of HCC patients were downloaded from NCBI with the search number GSE242889, including three patients with MVI present and two patients with MVI absent. None of the patients had significant metastasis, and they had not yet been treated. The downloaded scRNA-seq data were loaded into the “Seurat” package (Butler et al., 2018) and read using the Read10X function. Only cells with the proportion of mitochondrial genes <25% and the number of genes between 200 and 5000 were intercepted and normalized using the SCTransform function. The “Harmony” package (Korsunsky et al., 2019) was used for the elimination of batch effects between samples. The RunPCA function was used to perform principal component analysis (PCA) in Seurat, selecting the top 20 principal components, and the FindNeighbors and FindClusters functions were used to conduct cell clustering. According to the markers in the CellMarker2.0 database, the cell identity was identified and projected on UMAP. The cell identity was identified according to the markers contained in the CellMarker2.0 database (Hu et al., 2023) and projected on the UMAP.

### Differential expression analysis based on scRNA-seq data

The FindAllMarkers function was used to perform differential expression analysis between determined cell clusters in Seurat. For differentially expressed genes (DEGs), it must be in accordance with only.pos = TRUE, min.pct = 0.25, logfc.threshold = 0.25, and p < 0.05 (Xu et al., 2024).

### Gene Ontology (GO) functional enrichment analysis

Specific genes were uploaded to the DAVID database (Dennis et al., 2003; Wang et al., 2025), which can correlate genes from the input list to biological annotations. The Functional Annotation Chart was selected in Shortcut to DAVID Tools to perform GO functional enrichment analysis, and the annotated results were presented in the form of bar charts.

### Analysis of intercellular communication

The CellChat package (Jin et al., 2021) is a versatile and easy-to-use toolkit for inferring, analyzing, and visualizing intercellular communication from any given scRNA-seq data. The CreateCellChat function was applied to create objects in CellChat, while identifyOverExpressed Genes and identifyOverExpressedInteractions were employed to identify overexpressed ligand–receptor (LR) pairs of cell subsets. The projectData function projected gene expression data onto the protein–protein interaction (PPI) network. The communication probability was calculated by the computeCommunProb function and displayed as a bubble plot by the netVisual_bubble function.

### Cell trajectory analysis

Monocle 2 (Qiu et al., 2017) inferred the evolution of cells during development by conducting pseudotime analysis based on the changes in gene expression levels of cell subsets over time. In brief, the newCellDataSet function was used to construct the objects, and the highly expressed DEGs in the cell cluster were calculated by the FindAllMarkers function and submitted to reduceDimension for dimensionality reduction. Monocle 2 generated a tree-like structure after completing pseudotime modeling.

### Calculation of the activity degree of specific gene sets in cells

The gene lists of angiogenesis and TGFβ signaling pathways in hallmark were downloaded from the MsigDB database and entered into AUCell together with the single-cell expression matrix to calculate the enrichment scores of gene subsets in each cell, with higher scores indicating higher enrichment of gene subsets in the cell.

### Acquisition of RNA-seq data

The TCGA-HCC cohort was selected from the public cancer genomics resource UCSC Xena to download the gene expression data of HCC genomes, and the gene expression value log2(FPKM+1) was obtained. The cohort also provided clinical follow-up data of HCC, including TNM stage and survival data, which were also included in the analysis.

### Cell culture and intervention

Human liver immortalized cells THLE-2 (C5664) and HCC cell lines Huh-7 and HepG2 were all purchased from Baidi Biotech Ltd. (Hangzhou, China) and BeiNa Culture Bio (Xinyang, China) and cultured in Roswell Park Memorial Institute-1640 medium (90023, Solarbio LifeSciences, Beijing, China) and high-glucose Dulbecco’s modified Eagle’s Medium (C3260, Solarbio LifeSciences). The culture media were additionally supplemented with 10% bovine calf serum (S9020, Solarbio LifeSciences) and 1% penicillin–streptomycin (P1400, Solarbio LifeSciences). All cells were authenticated via short tandem repeat (STR) genotyping recently, tested negative for *mycoplasma* contamination, and cultured in an incubator (3311, ThermoFisher, Waltham, MA) at 37°C with 5% CO_2_.

For the knockdown assay, the small interfering RNA specific to *CAMK2N1* and the control small interfering RNA were all synthesized and purchased from GenePharma (Shanghai, China). Thereafter, the Lipofectamine 2000 transfection reagent (11668027, ThermoFisher) was applied for the transfection as per the manuals provided by the manufacturer. All cells were harvested after 48 h and used in the subsequent assays. The target sequences for small interfering RNA specific to *CAMK2N1* are 5′-CGG​GTT​GTT​ATT​GAA​GAT​GAT​AG-3′ (si-*CAMK2N1*#1) and 5′-GGG​TTG​TTA​TTG​AAG​ATG​ATA​GG-3′ (si-*CAMK2N1*#2).

### Scratch test

The scratch test was adopted to evaluate the migration of HCC cells. In detail, transfected HCC cells in these two groups were seeded in a 6-well plate at a density of 1 × 10^5^ cells/well and received a scratch on the monolayer via a 10-μL pipette tip once they grew fully confluent. After culturing for 48 h, the cell debris was removed, and the cell residue was visualized under the optical microscope (DP27, Olympus, Tokyo, Japan). The wound closure (%) was accordingly calculated to reflect the migration status of cells in both groups.

### Transwell assay

For the Transwell assay to determine the invasion of HCC cells, a Transwell system with a pore size of 8 μm (3422, Corning, Inc., Corning, NY) was applied. Specifically, the upper chamber was filled with HCC cells and the non-serum culture media (final volume: 200 μL) and coated with matrix gel (M8370, Solarbio LifeSciences) in advance. Thereafter, HCC cells were routinely cultured for 48 h, following which the uninvaded cells in the upper chamber were removed carefully with a cotton swab. Those cells that migrated to the lower chamber were accordingly fixed in 4% fixative (P1110, Solarbio LifeSciences) for 15 min and stained using 0.1% crystal violet (G1063, Solarbio Lifesciences) for 15 min. All cells were also visualized under the same optical microscope used for the scratch test (Zhang et al., 2024).

### Reverse-transcription quantitative PCR

All cellular total RNA was extracted with TRIzol reagent (15596026, ThermoFisher), and the concentration was determined in a spectrophotometer (ND-2000, ThermoFisher). Subsequently, the complementary DNA was synthesized with a relevant assay kit (6110A, Takara, Shiga, Japan) and applied to the PCR process. A specific assay kit (204243, Qiagen, Hilden, Germany) and the ABI7500 thermocycling system (ThermoFisher) were both used to run the PCR at these parameters: 95°C for 15 min, followed by 94°C for 15 s, 60°C for 30 s, and 72°C for 30 s for 40 repeated cycles. The mRNA levels were finally calculated based on formula 2^−ΔΔc(t)^ with GAPDH as the reference gene (Livak and Schmittgen, 2001).

The primers applied are:

*CAMK2N1*: forward: 5′-GGA​CAC​CAA​CAA​CTT​CTT​CGG​C-3′; reverse: 5′-GTC​GGT​CAT​ATT​TTT​CAG​CAC​GTC-3′

GAPDH: forward: 5′-GTC​TCC​TCT​GAC​TTC​AAC​AGC​G-3′; reverse: 5′-ACC​ACC​CTG​TTG​CTG​TAG​CCA​A-3′

### Statistical analysis

All statistical analysis and visualization were performed using R software (version 4.3.1) and GraphPad Prism software (version 8.0.2). The difference of continuous variables between the two groups was tested by the Wilcoxon rank sum test, analysis of variance and Student’s t-test, and the correlation was measured by Pearson correlation analysis. Grouping before survival analysis was based on the median of gene expression values, and survival differences were calculated by the log-rank test. The threshold of statistical significance was set at *p* < 0.05 for statistical tests without a specified p-value.

## Results

### Cellular heterogeneity between HCC patients with MVI absent and MVI present

**FIGURE 1:**
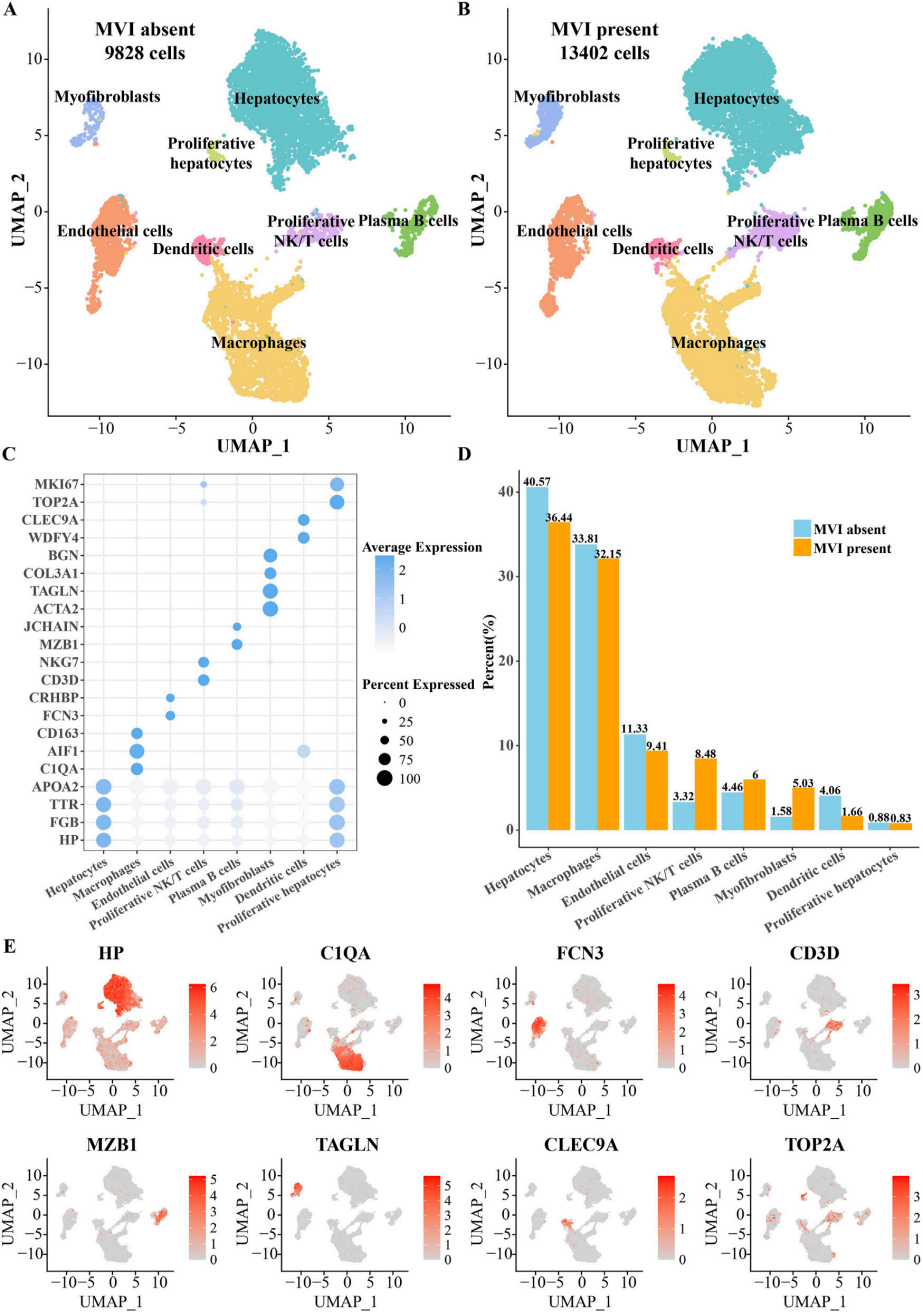
Cellular heterogeneity between HCC patients with MVI absent and MVI present. **(A)** UMAP diagram of cells in MVI-absent patients after clustering and annotation. **(B)** UMAP diagram of cells in MVI-present patients after clustering and annotation. **(C)** Dot plot of the DEG expression in each type of cell. **(D)** Percentage of each type of cell in HCC patients with MVI absent and MVI present. **(E)** Cell type-specific gene expression patterns.

Cluster analysis and annotation of 9,828 cells in HCC patients with MVI absent and 13,402 cells in HCC patients with MVI present identified eight major cell types: they were myofibroblasts (MFs), hepatocytes, proliferative hepatocytes, endothelial cells, dendritic cells, proliferative NK/T cells, plasma B cells, and macrophages (Figures 1A,B). Apolipoprotein APOE, transporter TTR, and the acute-phase proteins fibrinogen β (FGB) and haptoglobin (HP) were significantly overexpressed in both hepatocytes and proliferative hepatocytes (Dayoub et al., 2017). However, proliferative hepatocytes specifically expressed the proliferation-related markers *MKI* and *TOP2A* relative to proliferative hepatocytes. *CD163*, *AIF1*, and *C1QAM2* were significantly overexpressed in macrophages, all of which were related markers of M2 macrophages (Zhu et al., 2022). The high expression of *NKG7* and *CD3D* specifically distinguishes proliferative NK/T cells from other cells. The DEGs for identifying plasma B cells were *JCHAIN* and *MZB1* (Hensvold et al., 2023). The identity of MFs was confirmed based on the expression levels of *BGN*, *COL3A1*, *TAGLN*, and *ACTA2*. Dendritic cells specifically expressed *CLEC9A* and *WDFY41*, which are essential components for antigen cross-presentation in these cells (Ohara and Murphy, 2023) (Figures 1C,E). The largest difference in the proportion between MVI-absent patients and MVI-present patients was MFs, which accounted for a higher proportion in MVI-present patients (Figure 1D). This abnormal distribution of cells sent a message that MFs may play a role in the MVI of patients with HCC.

### Classification and characterization of MFs in HCC patients with MVI present

**FIGURE 2:**
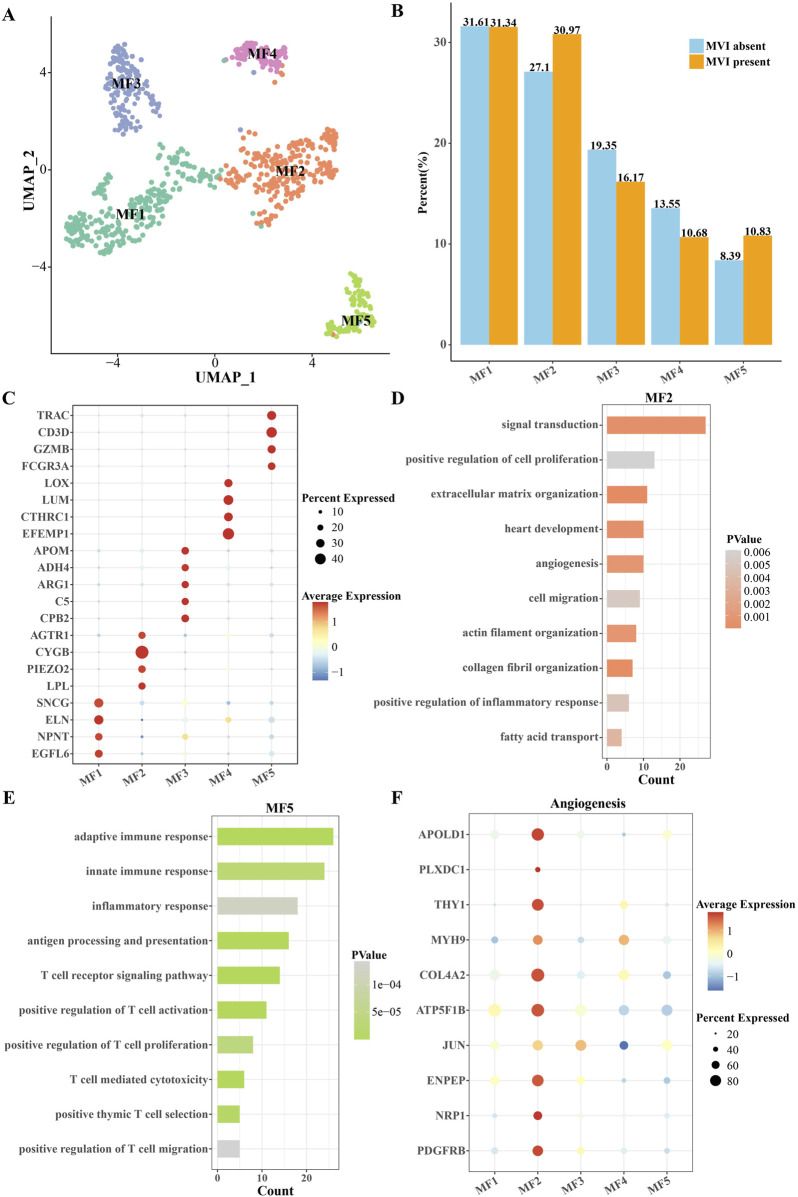
Classification and characterization of MFs in HCC patients with MVI present. **(A)** Cluster UMAP diagram of MFs. **(B)** Distribution of proportions of the five clusters of MFs in MVI-absent and MVI-present patients. **(C)** The dot plot shows the mean expression level of DEGs for each MF cluster. **(D)** Biological processes that were significantly enriched for highly expressed DEGs in MF2 cells. **(E)** Biological processes that were significantly enriched for highly expressed DEGs in MF5 cells. **(F)** Expression levels of key genes of angiogenesis in each MF cluster.

Because of the large difference in the proportion of MFs between HCC patients with MVI absent and those with MVI present, we proceeded to characterize this population in detail, focusing on the subgroup and gene expression profiles, intercellular communication networks, and differentiation trajectories of this population. The MFs were further divided into five clusters by cell clustering (Figure 2A). The proportions of MF2 and MF5 cells were significantly higher in HCC patients with MVI present than in those with MVI absent (Figure 2B). MF2 cells specifically expressed liver fibrosis-related genes *AGTR1*, *CYGB*, *PIEZO2*, and *LPL*, and MF5 cells specifically highly expressed immune-related genes *TRAC*, *CD3D*, *GZMB*, and *FCGR3A* (Figure 2C). Among the annotated biological processes, MF2 cells were linked with signal transduction, positive regulation of cell proliferation, extracellular matrix organization, angiogenesis, and cell migration (Figure 2D). Meanwhile, MF5 cells could be linked with adaptive immune response, innate immune response, and inflammatory response (Figure 2E). Therefore, MF2 cells were closely related to the promotion of angiogenesis, while MF5 cells were mainly related to the recruitment and activation of immune cells. Angiogenesis is one of the important mechanisms in the formation of MVIs. Most of the angiogenesis key genes were overexpressed in MF2 cells and underexpressed in MF5 cells (Figure 2F).

### Potential LR pairs that mediate communication between MF2 cells and hepatocytes

**FIGURE 3:**
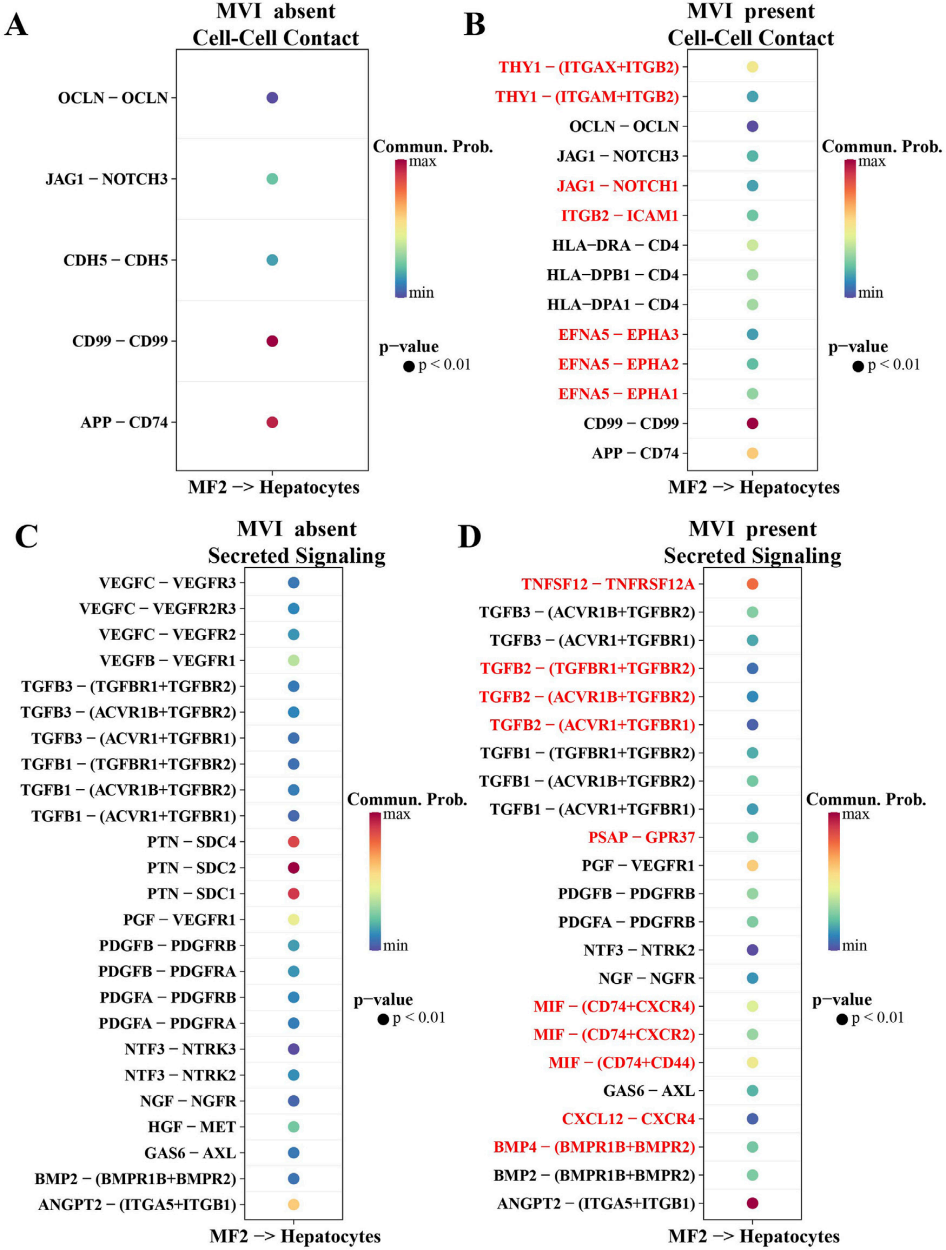
Potential LR pairs that mediate communication between MF2 cells and hepatocytes. **(A)** Direct contact type LR pairs that mediate communication between MF2 cells and hepatocytes in MV-absent patients. **(B)** Direct contact type LR pairs that mediate communication between MF2 cells and hepatocytes in MVI-present patients. **(C)** Secretory LR pairs mediating the communication between MF2 cells and hepatocytes in patients with MVI absent. **(D)** Secretory LR pairs mediating the communication between MF2 cells and hepatocytes in patients with MVI present.

By analyzing the LR pairs of communication between MF2 and hepatocytes in HCC patients with MVI absent and MVI present, it was found that direct contact LR pairs, including OCLN-OCLN, JAG1-NOTCH3, CDH5-CDH5, CD99^−^CD99, and APP-CD74, were involved in mediating the communication between MF2 cells and hepatocytes in both HCC patients with MVI absent and MVI present. The difference was that the communication between MF2 cells and hepatocytes in MVI-present patients was also mediated by THY1 (ITGAX + ITGB2), THY1- (ITGAM + ITGB2), JAG1-NOTCH1, ITGB2-ICAM1, EFNA5-EPHA3, EFNA5-EPHA2, and EFNA5-EPHA1 (Figures 3A,B). In addition to the direct contact type LR pairs, secretory LR pairs were also involved in the communication between MF2 cells and hepatocytes and were more complex in MVI-present patients. TNFSF12-TNFRSF12A, TGFB2-(TGFBR1+TGFBR2), TGFB2-(ACVR1B + TGFBR2), TGFB2-(ACVR1+TGFBR1), PSAP-GPR37, MIF- (CD74+CXCR4), MIF-(CD74+CXCR2), MIF-(CD74^+^CD44), CXCL12-CXCR4, and BMP4-(BMPR1B + BMPR2) were secreted LR pairs specifically involved in MF2 cells communication with hepatocytes in HCC patients with MVI present (Figures 3C,D).

### Pseudotime trajectories supported the promoting effect of MF2 cells on MVI formation

**FIGURE 4:**
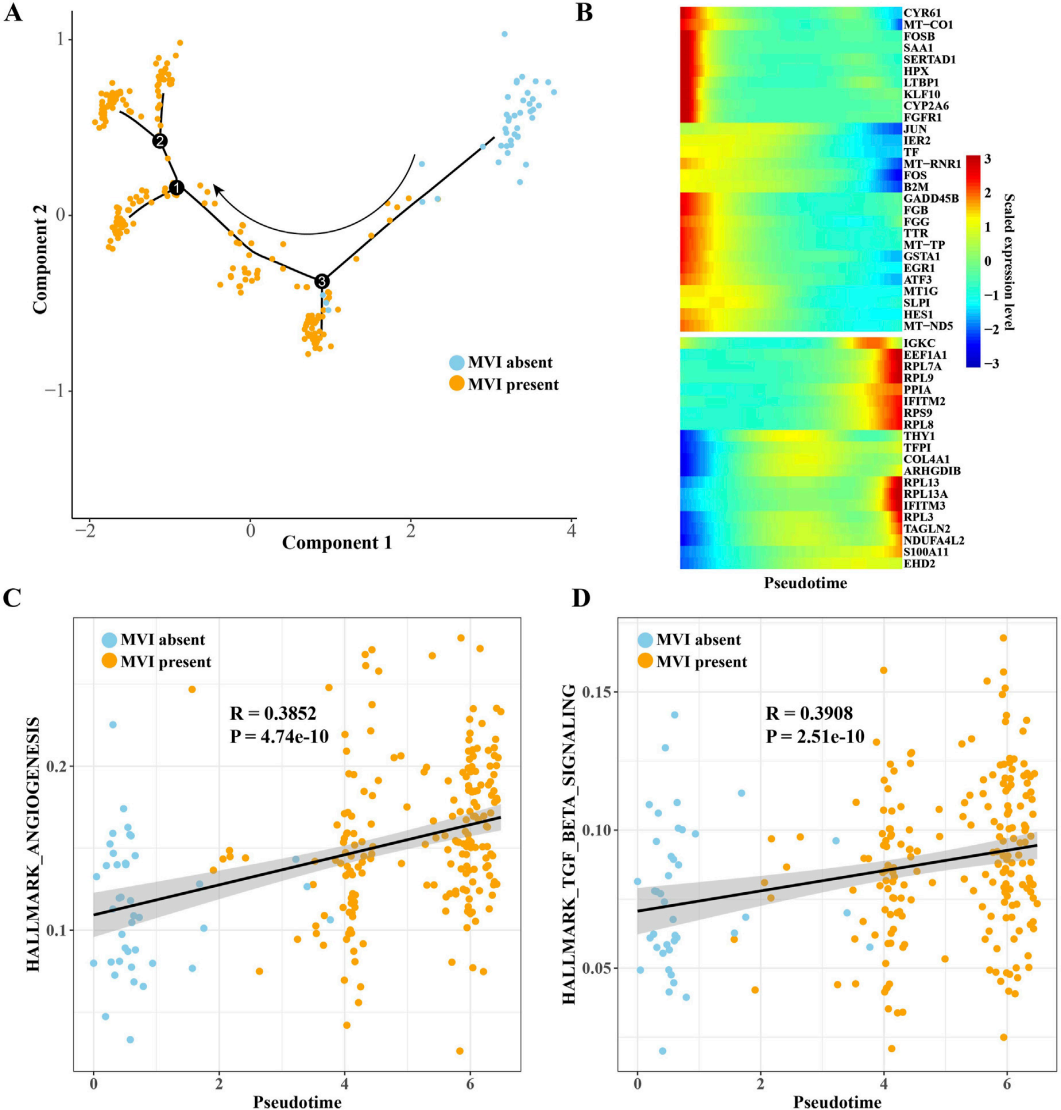
Pseudotime trajectories supported the promoting effect of MF2 cells on MVI formation. **(A)** The pseudotime trajectories of MF2 cells obtained by Monocle 2 analysis. **(B)** Genes resulting from Monocle 2 analysis that vary with pseudotime. **(C)** Pearson correlation between angiogenic gene sets and pseudotime. **(D)** Pearson correlation analysis between TGF-β pathway gene sets and pseudotime.

To characterize the dynamic changes in gene expression patterns of MF2 cells between MVI-absent and MVI-present samples, differentiation trajectories were constructed using Monocle 2. Most of the MF2 cells at the start of pseudotime were of MVI-absent origin, and MF2 cells of MVI-present origin appeared in pseudotime and were densely clustered at the end of the branch (Figure 4A). Monocle 2 also identified genes that varied with pseudotime (Figure 4B). The correlation between angiogenesis gene sets and TGF-β pathway gene sets was probed, and both angiogenesis and TGF-β pathway showed a significant positive correlation with pseudotime (Figures 4C,D). Therefore, it is speculated that MF2 cells may have a potential promoting effect on MVI formation.

### Genes used as HCC prognosis indicators were identified from MF2 cell markers

**FIGURE 5:**
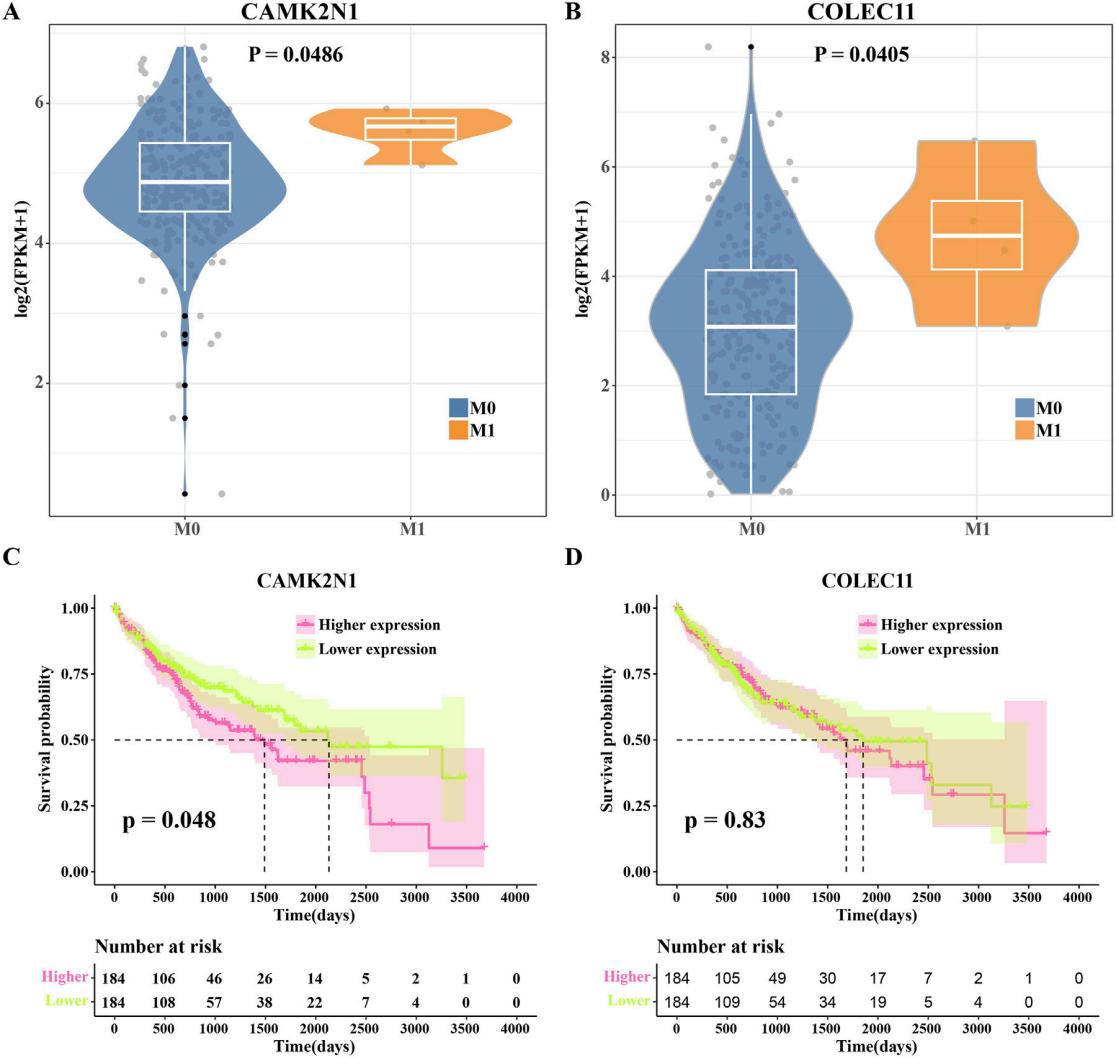
Genes used as HCC prognosis were identified from MF2 cell markers. **(A)** CAMK2N1 expression differences between M0 stage and M1 stage groups in the TCGA-HCC cohort. **(B)** COLEC11 expression differences between M0 stage and M1 stage groups in the TCGA-HCC cohort. **(C)** Kaplan–Meier curves obtained by analyzing the OS of patients in the TCGA-HCC cohort according to CAMK2N1 expression. **(D)** Kaplan–Meier curves obtained by analyzing the OS of patients in the TCGA-HCC cohort according to COLEC11 expression.

The expression of marker genes extracted from MF2 cells was detected in different M stages of the TCGA cohort. The expressions of *CAMK2N1* and *COLEC11* were significantly higher in M1 stage samples than in M0 stage patients (Figures 5A,B). The prognosis of patients in the TCGA cohort was predicted based on *CAMK2N1* and *COLEC11* expression. *CAMK2N1* expression showed a significant correlation with HCC prognosis as observed in the Kaplan–Meier curve. The overall survival (OS) of HCC patients with high expression was significantly shorter than that of patients with low expression (Figure 5C). However, no significant correlation between *COLEC11* expression and HCC prognosis was detected in the Kaplan–Meier curve obtained by analyzing OS based on *COLEC11* expression (Figure 5D). Therefore, *CAMK2N1* is a potential MF2 cell marker for predicting HCC prognosis.

### Cellular validation of HCC prognosis indicators in HCC cells

**FIGURE 6:**
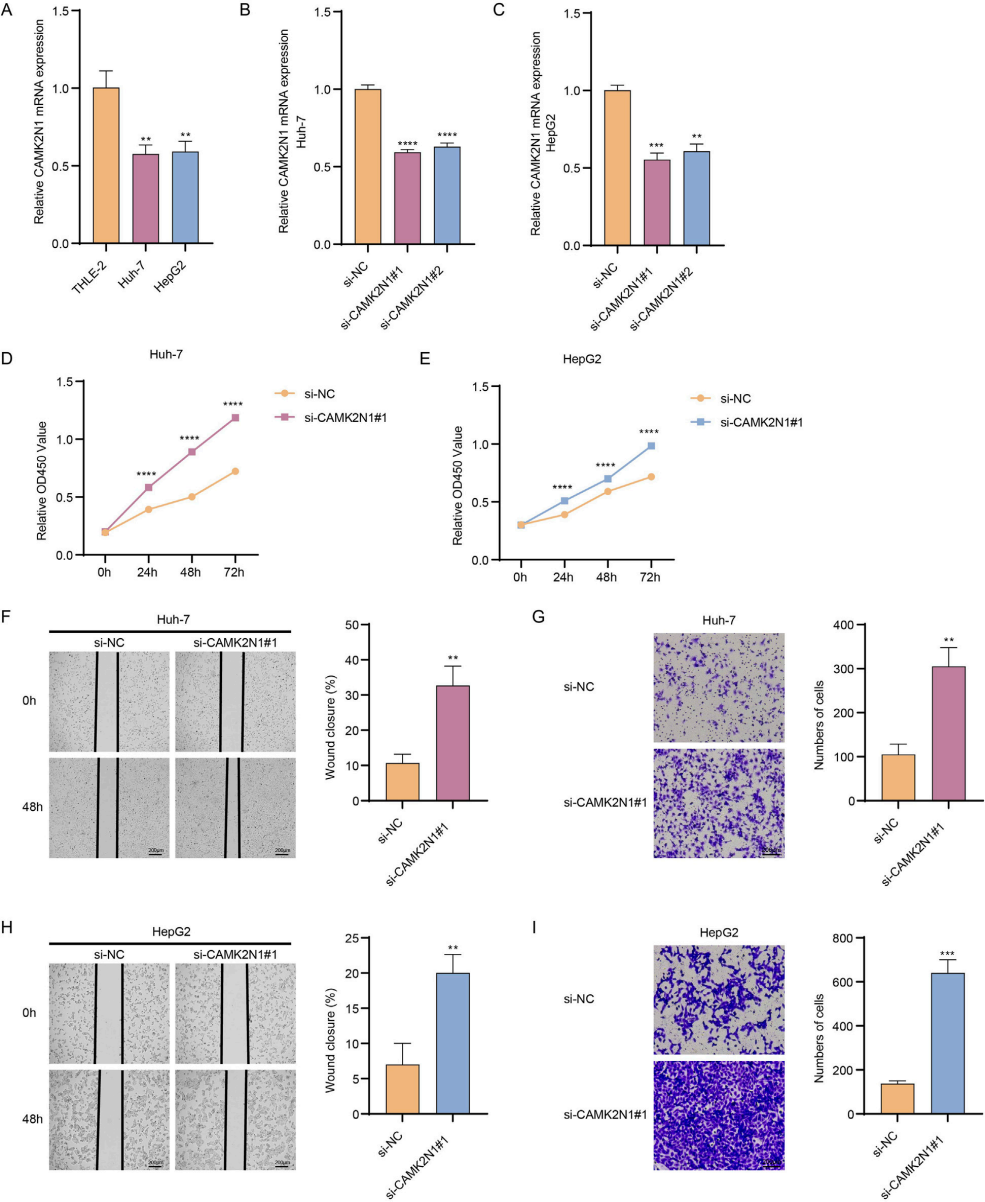
Cellular validation of HCC prognosis indicators in HCC cells. **(A)** *CAMK2N1* expression in both HCC cells (Huh7 and HepG2) and THLE-2 was assessed using reverse-transcription quantitative PCR. **(B,C)** Validation of the efficiency of *CAMK2N1* knockdown. **(D,E)** CCK-8 assay to evaluate the effect of *CAMK2N1* knockdown on the proliferation level of HCC cells. **(F,G)** Evaluate the effect of *CAMK2N1* gene knockout on cell migration and invasion levels based on Huh-7 cells. **(H,I)** Evaluate the effect of *CAMK2N1* gene knockout on cell migration and invasion levels based on HepG2 cells. Scale bar: 200 μm; ^**^ *p* < 0.01, ^***^ *p* < 0.001, and ^****^ *p* < 0.0001.

To explore the potential role of this *CAMK2N1* in HCC, the expression levels of these metrics were first calculated in HCC cells and control cells. We observed lower *CAMK2N1* expression in Huh-7 and HepG2 cells relative to THLE-2 cells (Figure 6A). In addition, to verify the potential effect of this gene on HCC cells, we validated its knockdown in two HCC cell lines (Figures 6B,C). The results of the CCK-8 assay showed that knockdown of the *CAMK2N1* gene significantly increased the proliferation level of Huh-7 and HepG2 cells (Figures 6D,E). Furthermore, *CAMK2N1* gene knockdown also significantly increased the migration and invasion levels of HCC cells (Figures 6F–I). These results indicated the scientific rationality of *CAMK2N1*, an MF2 cell marker gene significantly associated with HCC prognosis, as a potential prognostic biomarker.

## Discussion

MVI is defined by the invasion of cancerous cells into the small blood vessels encircling the liver tumor (Harding-Theobald et al., 2021). A meta-analysis shows that MVI-positive patients are twice as likely to relapse as MVI-negative patients (Chen et al., 2019). Microscopically, there is a broad spectrum of MVI (Hwang et al., 2023). To date, the clear mechanism of MVI in HCC has not been fully elucidated. It is generally accepted that the formation of MVI is a complex process with multi-step regulation, such as the pathological anatomy of cirrhosis, hemodynamics, and tumor molecular biology (Sun et al., 2023). This study studied the potential cell populations that promote the formation of MVI and the molecular networks that communicate with hepatocytes from the point of view of tumor cell biology and molecular biology, as well as candidate targets that can potentially indicate the prognosis of HCC. This study was the first to demonstrate (Chandarana et al., 2024): The cellular ecosystem of HCC was rich in hepatocytes, fibroblasts, and immune cells, including myofibroblasts, hepatocytes, proliferative hepatocytes, endothelial cells, dendritic cells, proliferative NK/T cells, plasma B cells, and macrophages (Wang et al., 2023). The largest proportion difference between MVI-absent patients and MVI-present patients was MFs, which were higher in MVI-present patients and were further divided into five MF subsets (Alawyia and Constantinou, 2023). Key angiogenesis genes were overexpressed in MF2 cells, and MF2 cells derived from MVI-present patients were enriched at the end of differentiation, which may potentially promote MVI formation. (Bansal et al., 2024). *CAMK2N1* was a potential MF2 cell marker for predicting the prognosis of HCC. These findings provide novel insights into the cellular and molecular basis of MVI formation in HCC, highlighting MF2 cells as a key pro-angiogenic stromal component that may serve as both a mechanistic driver and a prognostic indicator of vascular invasion.

HCC often occurs in the background of liver fibrosis, and the activation of hepatic stellate cells (HSCs) runs through the whole process of the development of HCC precancerous lesions (Shan et al., 2023; YOON et al., 2023). HSCs and their activated derivatives are commonly referred to as MFs (Bogomolova et al., 2024). The imbalance of MFs is associated with an increased risk of HCC in patients during HCC progression (Filliol et al., 2022). In the present study, we found that increased MFs in HCC were associated with MVI generation. MF2 is the major MF cluster that promotes the formation of MVI. It is known from previous reports that in TME, HCC cells secrete various soluble factors that are responsible for the phenotypic activation of quiescent HSCs. Tumor cells use activated HSC-derived extracellular matrix (ECM) for migration and invasion (Ezhilarasan and Najimi, 2023). In this study, we found that MF2 cells were significantly associated with positive regulation of cell proliferation, extracellular matrix organization, angiogenesis, and cell migration. Secretory and direct contact ligand–receptor (LR) pairs that mediate the communication between MF2 cells and hepatocytes in HCC patients with MVI present were also identified. Among them, JAG1-NOTCH3 ligand–receptor pairing is related to the regulation of phenotypic maturation of vascular smooth muscle cells (Zohorsky et al., 2021). HSC regulates the differentiation of HPC into hepatocytes through the TGF-β1/Jagged1 signal transduction axis (Aimaiti et al., 2019). The ITGB2-ICAM1 axis regulates ECM-related features (Li et al., 2023). Signaling between EFNA5 and EPHA is also involved in mediating biological processes such as angiogenesis and cancer (Irie et al., 2008). TNFSF12 and its receptor TNFRSF12A are involved in the inflammatory response associated with vascular remodeling (Mendez-Barbero et al., 2019). CXCL12/CXCR4 promotes ECM production characteristic of fibrosis and induces phenotypic transformation of myofibroblasts (Patalano et al., 2018). These studies provided favorable evidence that MF2 cells may interact with hepatocytes through these molecular interactions, thereby affecting the ECM or angiogenesis in HCC.

We extracted the marker genes of MF2 cells and identified them as HCC prognostic markers in the TCGA cohort. *CAMK2N1* was identified, which was highly expressed in high M-stage cancers and significantly correlated with poor prognosis of HCC. *CAMK2N1* has been recognized as a tumor suppressor that downregulates the β-catenin/c-Myc oncogenic signaling pathway. Accordingly, Tang et al. discovered that Circ-IP6K2 plays a role in hindering the advancement of renal cell carcinoma by influencing the miR-1292-5p/*CAMK2N1* axis (Tang et al., 2024). In addition, N6-methyladenosine-induced miR-182-5p was also able to promote multiple myeloma by regulating *CAMK2N1*, which in turn promotes multiple myeloma (Bao et al., 2024). In previous studies, increased *CAMK2N1* expression has shown an association with increased prostate cancer aggressiveness (Carneiro et al., 2019). However, Peng et al. showed that *CAMK2NA* is a 1p36 tumor suppressor gene, and silencing and ectopic expression of *CAMK2N1* enhanced and inhibited cell proliferation, colony formation, and xenograft tumor growth, respectively, in HCC nude mice (Peng et al., 2021). We suspect that the reasons for these differences include tumor heterogeneity and differences in the number of samples studied.

Our study has some limitations. For example, the scRNA-seq data used in this study had a small sample size and may have inter-individual biological differences, limiting the generalizability of conclusions on the association between cell subpopulation proportions and MVI. In the future, more independent cohorts and multi-center scRNA-seq data can be combined for integrated analysis to enhance the robustness and broad applicability of the results. Meanwhile, the ability to analyze cell distribution and heterogeneity can be further enhanced by spatial transcriptome technology. In addition, this study focuses on the effect of the MF2 cell subpopulation on MVI, and although the strategy is clearly focused, the synergistic effect of other microenvironmental cells on MVI formation may be missed. For this reason, subsequent studies will combine cellular communication networks, jointly analyze other key cell subpopulations, and construct a model of MVI formation driven by multi-cell interactions to more comprehensively understand the synergistic mechanisms in the tumor microenvironment. Finally, although the study revealed major cell subpopulations, cell types of potential functional significance in the tumor microenvironment were not stably identified due to technical limitations or sparse data, and some important regulatory networks may have been missed. Therefore, we will further optimize the tissue processing and library construction strategies to enhance the capture of rare cell populations and combine protein markers to improve the accuracy of cell identification.

## Conclusion

In summary, this study reported cellular heterogeneity between HCC patients with MVI absent and MVI present, demonstrated the role and underlying mechanism of MFs in promoting MVI formation, and provided CAMK2N1 as a target in MF markers for HCC prognosis.

## Data Availability

The datasets presented in this study can be found in online repositories. The names of the repository/repositories and accession number(s) can be found in the article/supplementary material.

## Abbreviations

DEG, differentially expressed gene; GO, Gene Ontology; HCC, hepatocellular carcinoma; MF, myofibroblast; MVI, microvascular invasion; PCA, principal component analysis; PPI, protein–protein interaction; ScRNA-seq, single-cell RNA sequencing; TCGA, The Cancer Genome Atlas.
